# Neuroimaging insights into lung disease-related brain changes: from structure to function

**DOI:** 10.3389/fnagi.2025.1550319

**Published:** 2025-02-20

**Authors:** Miao He, Yubo Liu, Zhongtian Guan, Chunlin Li, Zhixi Zhang

**Affiliations:** 1 School of Biomedical Engineering, Capital Medical University, Beijing, China; 2 Aerospace Information Research Institute, Chinese Academy of Sciences, Beijing, China

**Keywords:** lung-brain axis, neuroimaging, lung disease, MRI-based brain changes, neurological impact of lung disease

## Abstract

Lung diseases induce changes in brain structure and function, leading to a range of cognitive, emotional, and motor deficits. The concept of the lung-brain axis has been proposed through neuroanatomy, endocrine, and immune pathway, while a considerable number of studies also explored the existence of the lung-brain axis from a neuroimaging perspective. This survey summarizes studies exploring the relationship between lung disease and brain structure and function from neuroimaging perspective, particular in magnetic resonance imaging (MRI). We have collated existing lung diseases studies and categorized them into four types: chronic obstructive pulmonary disease (COPD), coronavirus disease 2019 (COVID-19), lung cancer and other lung diseases. The observed structural and functional changes in the brain and cognitive dysfunction induced by lung diseases are discussed. We also present distinct pattern of brain changes in various lung diseases. Neuroimaging changes in COPD are concentrated in the frontal lobes, including gray matter atrophy, white matter damage, and reduced perfusion. Patients with COVID-19 exhibit extensive microhemorrhages and neuroinflammation, brain regions functionally connected to the primary olfactory cortex show greater changes. For lung cancer patients, brain changes are mainly attributed to the neurotoxicity of radiotherapy and chemotherapy, with damage concentrated in subcortical structures, patients with cancer pain demonstrate hyperconnectivity in motor and visual networks. The survey also discusses the pathological mechanisms revealed in neuroimaging studies and clinical significance of current studies. Finally, we analyzed current limitations, mainly in terms of small sample size, non-standardized criteria, reliance on correlation analyses, lack of longitudinal studies, and absence of reliable biomarkers. We suggest future research directions should include leveraging artificial intelligence for biomarker development, conducting longitudinal and multicenter studies, and investigating the systemic effects of lung disease on the brain and neuromodulation strategies.

## 1 Introduction

The central nervous system (CNS) and respiratory system are intricately interconnected through the autonomic nervous system. This connection facilitates reflexive responses like coughing and sneezing in asthma and chronic obstructive lung disease (COPD) (De Virgiliis and Di Giovanni, 2020). The respiratory system also impact the CNS via CO_2_-sensitive brainstem nuclei to regulate cortisol level (Abelson et al., 2010). Neuroendocrine lung tumors influence central neuroendocrine function by releasing endocrines like adrenocorticotrophic (Gandhi and Johnson, 2006). Immune responses in the lungs, including autoreactive T cell proliferation, can lead to autoimmune effects, and lung microbiome may regulate the susceptibility of CNS to autoimmune diseases (Li et al., 2023; Hosang et al., 2022). Evidences from neuroanatomy, endocrine, immune pathways support that lung pathology can impact brain structure and function. Exploring the brain changes in lung diseases may reveal new avenues for lung disease monitoring and targeted treatment strategies. While many studies have explored the impact of lung diseases on the brain using neuroimaging techniques. This survey integrates these studies to explore the multifaceted effects of lung diseases on brain structure and function, analyze related mechanisms, and summarize the clinical significance and future research directions.

Advanced neuroimaging techniques can visualize the impact of lung diseases on brain structure and function. These techniques include structural magnetic resonance imaging (MRI), diffusion tensor imaging (DTI), resting-state functional MRI (rs-fMRI), perfusion MRI and positron emission tomography (PET). The non-invasive, high resolution and multimodal capabilities of neuroimaging technologies render them powerful tools for investigating lung-brain interactions. Each modality offers unique advantages and limitations. Structural MRI can capture anatomical details. It is widely used to assesses gray matter (GM) atrophy, cortical morphology changes and white matter (WM) changes in COPD, coronavirus disease 2019 (COVID-19), lung cancer, and other lung diseases (Esser et al., 2016; Chen et al., 2016; Qin et al., 2020; van der Knaap et al., 2024; Parsons et al., 2021; Douaud et al., 2022; Simó et al., 2016a; Simó et al., 2016b; Liu et al., 2022c; Hopkins et al., 2006; Roy et al., 2021; Mohammadi-Nejad et al., 2022; Vandiver et al., 2022). However, it cannot detect WM microstructural changes. DTI detects WM microstructural damage by measuring fractional anisotropy (FA) and other diffusion metrics in COPD and lung cancer patients (Lee S. et al., 2019; Simó et al., 2016b; Simó et al., 2016a; Liu et al., 2022c). Rs-fMRI reveals altered functional connectivity (FC) in the default mode network (DMN) in asthma (Zhang et al., 2017; Wang et al., 2023; Zhu et al., 2023; Wu et al., 2023), and alterations in brain network topology and abnormal FC in pain perception circuits (Chammah et al., 2021; Hu et al., 2022; Wei et al., 2024; Zhou X. et al., 2022). Rs-fMRI can assess brain activity, but depend on data preprocessing and subject conditions. Perfusion MRI offers insights into cerebrovascular dynamics, implicating vascular pathology in long-term neurological sequelae of post-COVID-19 patients (Ajčević et al., 2023; Mohammadi and Ghaderi, 2024). PET imaging elucidates metabolic and neurotransmitter alterations in lung diseases (Fontana et al., 2020; Golan et al., 2009). But PET has lower spatial resolution compared to MRI and needs radioactive tracers. These neuroimaging techniques complement each other and are often combined to provide a more comprehensive understanding of lung-brain axis.

This survey summarizes neuroimaging findings across various lung diseases. It focuses on brain structural and functional changes caused by these conditions. It aims to clarify the neuroimaging mechanisms underlying lung-brain interactions using multimodal imaging. The survey assesses the clinical relevance of these findings. These insights lay a foundation for advancing the diagnosis and treatment of multisystem diseases.

**FIGURE 1:**
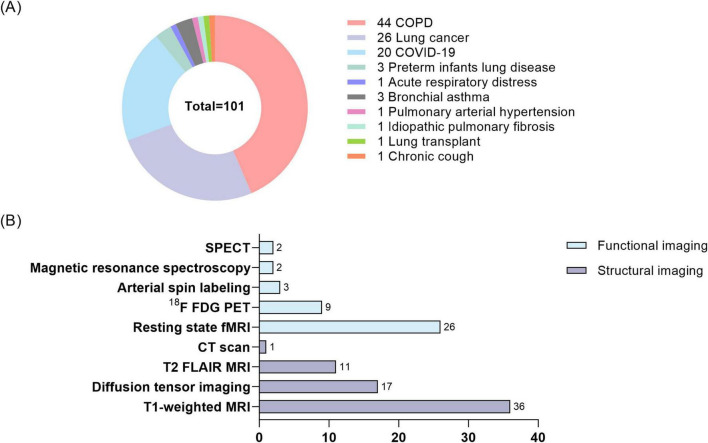
Distribution of lung disease studies and number of neuroimaging techniques. **(A)** Percentage of lung diseases studied in lung-brain interaction studies. **(B)** Number of neuroimaging modalities employed in lung-brain interaction studies. COPD, chronic obstructive lung disease; COVID-19, coronavirus disease 2019; SPECT, single-photon emission computed tomography. ^18^F-FDG-PET, ^18^F-FDG positron emission tomography.

**FIGURE 2:**
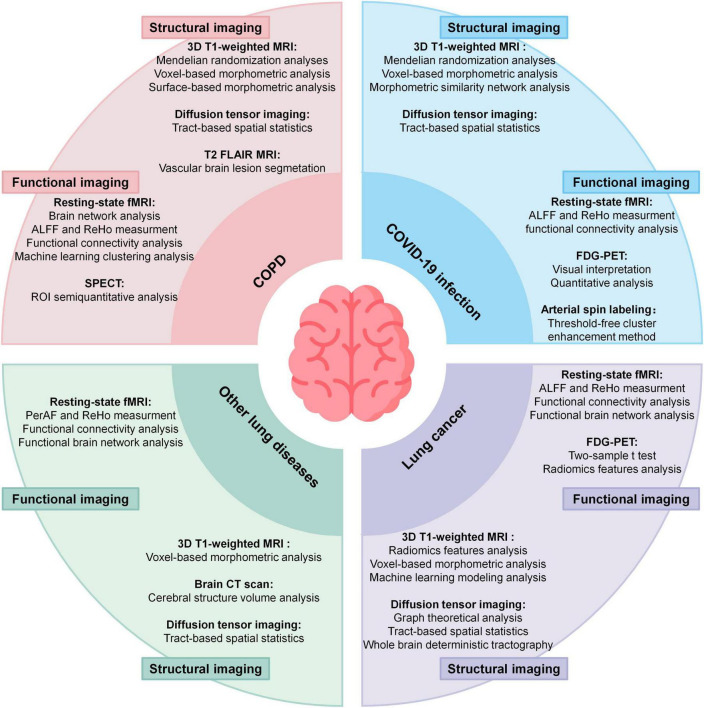
Summary of neuroimaging methods applied in 4 kind of lung disease and corresponding analyses methods. ALFF, amplitude of low-frequency fluctuations; ReHo, regional homogeneity; PerAF, percent amplitude of fluctuation; SPECT, single-photon emission computed tomography. ^18^F-FDG-PET, ^18^F-FDG positron emission tomograph.

Figure 1A highlights four major lung diseases studied in this field. Among these, COPD is the most extensively investigated condition, followed by lung cancer, COVID-19, and other less-explored conditions such as asthma and pulmonary artery hypertension. Figure 1B summarizes the neuroimaging modalities employed in current studies, categorizing them into structural and functional imaging techniques. Figure 2 builds on the categorization of lung diseases in Figure 1A, outlining the neuroimaging methods and associated data analysis approaches applied in studies of these conditions. Table 1 summarizes studies associating lung function indices with neuroimaging, and lists the main findings.

**TABLE 1:** List of research that found associations between lung function indicators and brain changes.

Lung function indices	Neuroimaging	Researchers	Methodology	Main findings
COPD anxiety assessments	T1-weighted MRI	Esser et al., 2016	VBM analyses to measure cortical degeneration. Correlation analyses to assess the relation between cortical degeneration and COPD specific fear.	Reduced GM in anterior cingulate is associated with breathlessness and disease duration.
Arterial oxygen saturation (SO_2_)	T1-weighted MRI	Chen et al., 2016	SBM to obtain cortical parameters. Demographic, physiological, and cognitive assessments were correlated with cortical indices.	Reduced parieto-frontal cortical thickness correlates with visuospatial impairment, thinner dorsolateral prefrontal cortex links to lower SO_2_.
Predicted forced vital capacity (FVC), predicted forced expiratory volume in one second (FEV1)	T2 FLAIR MRI	Xiao et al., 2022	Cross-sectional and longitudinal correlation analyses of lung function with cognition and cross-sectional correlation analyses of lung function with vascular brain lesions.	COPD and restrictive lung function patients show impaired global cognitive function and higher prevalence of lacunar infarcts.
FEV1, FVC	T1-weighted MRI	Zhou L. et al., 2022	The association between lung function and brain MRI biomarkers related to cognitive function was analyzed using Cox proportional hazards regression model.	Participants with lung function impairments had a higher risk of dementia. MRI indices supported adverse effects and provided insight into potential pathophysiology biomarkers.
Partial pressure of oxygen (PaO_2_)	T1-weighted MRI	Zhang et al., 2013; Zhang et al., 2012	VBM analyses to measure GM volume. Correlation analysis the relationship between PaO_2_ and GM volume.	In COPD patients, GM volume in the rectus, orbital gyrus, orbital gyrus, insula, cingulate gyrus, thalamus, and amygdala positively correlates with arterial blood PaO_2_.
FEV1, FVC	Structure MRI	Spilling et al., 2021	Brain MRI markers of brain structure were tested for association with disease markers in other organs.	Multivariable regression model showed that less organized white matter microstructure was associated with lower respiratory function
FEV1	T1-weighted MRI DTI	Yin et al., 2019	VBM and TBSS identified GMD and white matter changes in COPD. Correlation analyses between imaging parameter changes and cognitive and pulmonary functional impairments	In the COPD group, frontal gyrus GMD positively correlates with MoCA scores and FEV1, while corpus callosum MD/RD and superior corona radiata AD negatively correlate with both.
FEV1, FVC, FEV1/FVC	Structure MRI	Fang et al., 2024	Mendelian randomization study to explore the causal relationship between COPD, lung function, and cortical structure.	COPD and lung function indices causally affect brain regions like the pars orbitalis, cuneus, and inferior parietal gyrus.
FEV1, FVC, FEV1/FVC	T2 FLAIR MRI	Takamatsu et al., 2021	WMH was quantified on brain MRI images using a morphometric program. AL was defined as FEV1/FVC < 0.70.	AL is associated with increased WMH volume and with more frequent localization of WMH in the frontal lobe.
Forced expiratory volume 1% (FEV 1%), FVC, FEV1, Arterial blood gases	TMS	Mohamed-Hussein et al., 2007		TMS parameters significantly correlated with FVC, FEV1, FEV1% and arterial blood gases (pH, PaO_2_, HCO3).
FEV1% pred PaO_2_	Rs-fMRI	Lu et al., 2019	Voxel-based *t*-tests identified ALFF differences between COPD patients and controls. Additional fMRI with oxygen delivery examined the effects of elevated PaO_2_ on ALFF in both groups.	COPD patients show reduced ALFF in the basal ganglia and right thalamus, correlated with PaO_2_ and FEV1% pred. Oxygen increased ALFF in controls but not in COPD.
FEV1, FVC, FEV1/FVC	Rs-fMRI	Tang et al., 2022	Rs-fMRI were analyzed using ICA to identify resting-state networks (RSNs). sFNC and dFNC were constructed, and FC differences between COPD patients and healthy controls were compared.	dFNC properties, including mean dwell time and fractional windows, significantly correlate with clinical indicators like FEV1, FEV1/FVC in COPD patients.
Inspiratory load	Rs-fMRI	Yu et al., 2016	Functional connectivity and Granger causality of the respiratory network were analyzed at rest and during inspiratory loading in COPD and controls.	COPD patients show reduced motor cortex FC with the contralateral cortex and no brainstem connection. FC correlates with lung function and severity, suggesting brain activity modulation to enhance motor cortex FC and respiratory muscle performance.
FEV 1%, FEV1/FVC, PaO_2_, PaCO_2_, SaO_2_	Rs-fMRI	Xin et al., 2019	Rs-fMRI evaluated local brain signal synchrony. Correlations between ReHo and neuropsychological scores in COPD patients were analyzed using Pearson’s correlation.	COPD patients had reduced ReHo in occipital lobe, lingual, precuneus, and precentral gyrus. ReHo in precuneus correlated positively with lung function and orientation function, but negatively with PaCO_2_.
FEV1, FEV1/FVC	Rs-fMRI	Li et al., 2020	Intrinsic functional hub changes were analyzed via DC, with FC assessing connectivity to the brain, and correlations linking abnormal DC to clinical variables.	COPD patients showed reduced DC in the LG, SMA, and PCL. Decreased DC in the PCL correlated with FEV1 and FEV1/FVC.
FEV1	transcranial Doppler ultrasound	Hlavati et al., 2019	Baseline CVR of MCA and BA were analyzed using transcranial Doppler ultrasound and BHI.	FEV1 positively correlates with CVR. COPD patients show reduced CVR, worsening with airflow limitation severity.
Predicted FVC, Predicted FEV1	FDG-PET	Son et al., 2023	Lung function was analyzed alongside FDG-PET uptake in cortical and cognition-related regions.	FVC and FEV1 positively correlated with SUVRs across brain regions, with no significant differences between cognition-related and other areas.

VBM, voxel-based morphometry; SBM, surface-based morphometry; GM, gray matter; DTI, diffusion tensor imaging; TBSS, tract-based spatial statistics; GMD, gray matter density; MD, mean diffusivity; AD, axial diffusivity; RD, radial diffusivity; WMH, white matter hyperintensities; AL, airflow limitations; TMS, transcranial magnetic stimulation; rs-fMRI, resting-state fMRI; ALFF, amplitude of low-frequency fluctuations; ICA, independent component analysis; RSNs, resting-state networks; sFNC and dFNC, static and dynamic functional network connectivity; FC, functional connectivity; ReHo, regional homogeneity; DC, degree centrality; LG, lingual gyrus; SMA, supplementary motor area; PCL, paracentral lobule; CVR, cerebrovascular reactivity; MCA, middle cerebral artery; BA, basilar artery; BHI, breath-holding index.

The rest of this manuscript is organized as follows. Section “2 COPD and neuroimage changes” focuses on the impacts of COPD on brain structure, function, and cognition; Section “3 Neuroimaging changes due to lung cancer” discusses the effects of lung cancer on brain structure, function, and cognition; Section “4 COVID-19 and neuroimage changes” elaborates on the influence of COVID-19 on brain structure, function, and cognition; Section “5 Neuroimaging changes in other lung diseases” examines the effects of other lung diseases on brain structure and function; Section “6 Mechanisms of neuroimaging findings in lung diseases” analyze the potential mechanisms underlying neuroimaging findings in lung diseases; Section “7 Clinical significance of neuroimage research” summarize the clinical significance of neuroimaging studies; Section “8 Limitations of current research and future directions” discuss the limitations of current research and suggestions for future directions.

## 2 COPD and neuroimage changes

Growing evidence highlights the systemic impact of COPD. Chronic hypoxia and respiratory instability drive structural and functional disruptions in brain, link lung dysfunction to neurological decline.

### 2.1 Structural effects of COPD on the brain

**FIGURE 3:**
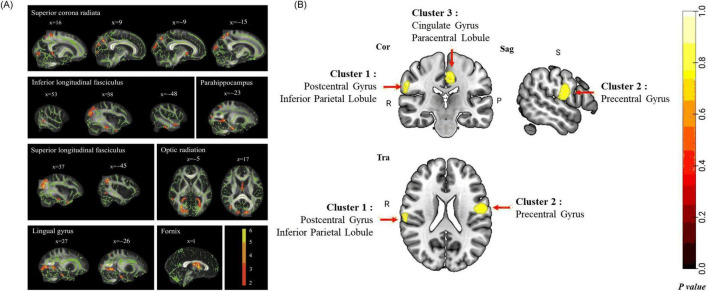
Cerebral structural changes on COPD patients. **(A)** Group comparison of fractional anisotropy (FA) value on tract-based spatial statistics (TBSS). COPD Patients show significantly lower FA value than healthy controls (*p* < 0.05) in superior corona radiata, inferior longitudinal fasciculus, superior longitudinal fasciculus, optic radiation, fornix, parahippocampus, and lingual gyrus (Zhang et al., 2012). **(B)** The pattern of GM abnormalities in COPD patients using voxel-based meta-analysis, results revealed significant GM abnormalities in the right postcentral gyrus (including inferior parietal lobule), left precentral gyrus, and left cingulate gyrus (including paracentral lobule) in COPD patients compared with HCs. Cor, coronal; Sag, sagittal; Tra, transverse; R, right; P, posterior; S, superior. Permutation test *P* < 0.05, FWE corrected (Liang et al., 2024).

**FIGURE 4:**
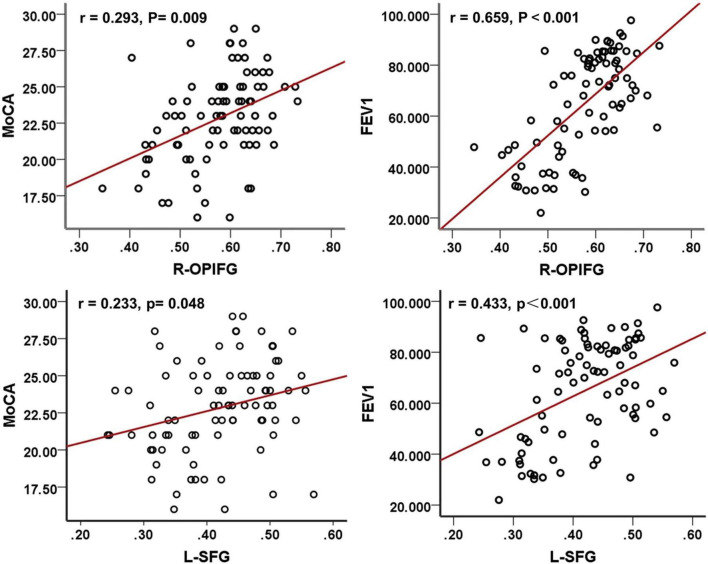
Correlation analyses among MoCA scores, FEV1, and GMD in COPD patients. MoCA, Montreal cognitive assessment; FEV1, forced expiratory volume in 1 s; COPD, chronic obstructive pulmonary disease; R-OPIFG, right orbital part of the inferior frontal gyrus; and L-SFG, left superior frontal gyrus (Yin et al., 2019).

Structural MRI studies in COPD patients reveal widespread brain abnormalities. These include reduced GM volume, WM integrity loss, WM hyperintensities (WMHs), and cortical thickness alterations. These structural changes involve multiple brain regions and are correlated with cognitive decline, motor dysfunction, and increased dementia risk (Xiao et al., 2022; Zhou L. et al., 2022). COPD-related GM atrophy predominantly affects the hippocampus, prefrontal cortex, cingulate gyrus, limbic system, and subcortical structures, along with cortical thinning in motor, parietal, prefrontal, Broca’s areas (Esser et al., 2016; Fukatsu-Chikumoto et al., 2024; Zhang et al., 2013; Zhang et al., 2012; Chen et al., 2016; Liang et al., 2024). A voxel-based meta-analysis further delineated the patterns of GM atrophy in COPD patients (Figure 3B). DTI analyses reveal decreased FA in the superior and inferior longitudinal fasciculi, corona radiata, optic radiations, and fornix, and altered diffusivity in the corticospinal tract, thalamus, and midbrain (Figure 3A; Dodd et al., 2012; Spilling et al., 2021; Lee S. et al., 2019; Zhang et al., 2012). Furthermore, COPD patients showed increased WMHs and enlarged perivascular spaces, with severity positively correlating with disease duration (Spilling et al., 2017; Qin et al., 2020). Voxel-based morphometry (VBM) and surface-based morphometry (SBM) analyses further identified GM atrophy in several brain regions. These include the anterior insula, thalamus, caudate, and para-hippocampal gyrus. Some changes correlate with cognitive performance (Montreal Cognitive Assessment, MoCA scores) and lung functions (forced expiratory volume in one second, FEV1) (Zhang et al., 2013; Yin et al., 2019; see Figure 4). Mendelian randomization studies suggest potential causal links between COPD and cortical atrophy in orbital, cuneate and inferior parietal gyrus (Fang et al., 2024). Structural damage to the corpus callosum, with reduced FA and increased mode of anisotropy, may underlie motor deficits and muscle weakness in COPD (Cabibel et al., 2020). Some studies reporting no evidence of GM atrophy in stable COPD patients. But progressive WM lesions in advanced disease stages emphasize the importance of early interventions to mitigate cognitive and neurological impacts (Spilling et al., 2017; Liang et al., 2024; Takamatsu et al., 2021; Yin et al., 2019).

### 2.2 Functional effects of COPD on the brain

**FIGURE 5:**
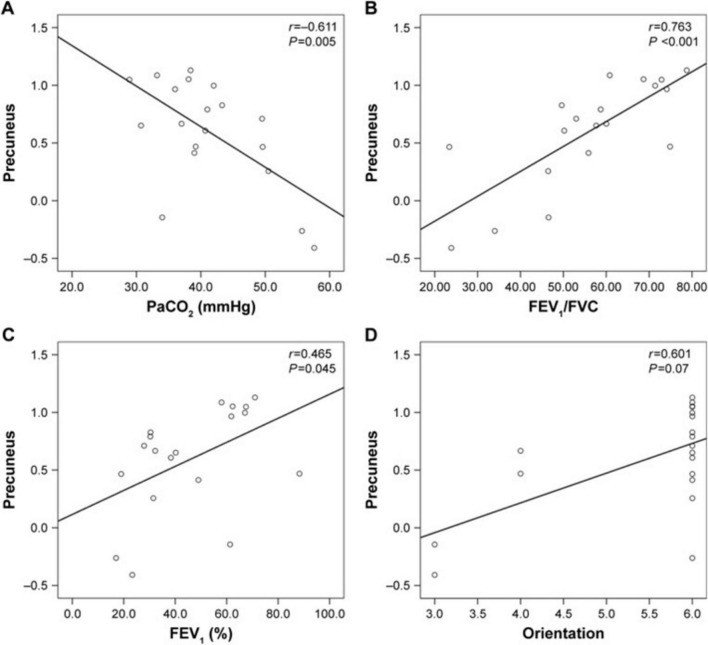
Correlation between the ReHo values in the precuneus and the clinical parameters **(A)** PaCO2 **(B)** FEV1/FVC **(C)** FEV1(%) **(D)** Orientation.

**FIGURE 6:**
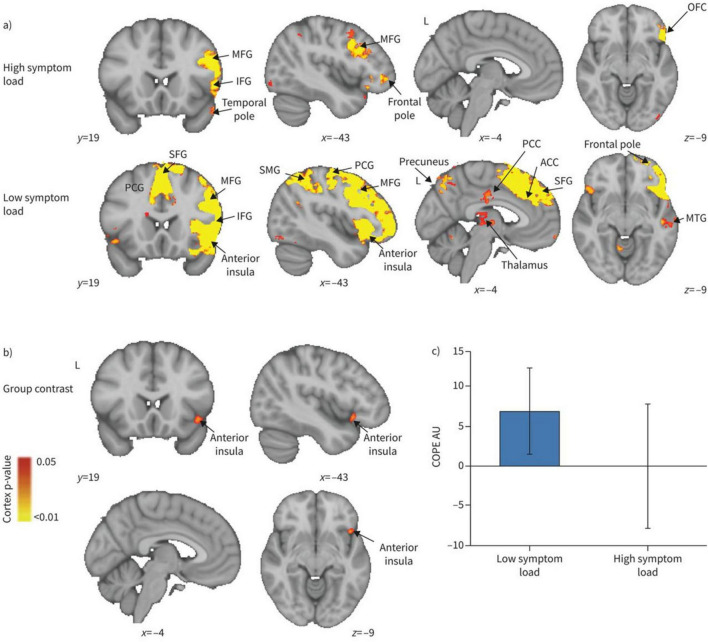
Blood oxygen level dependent activity in response to breathlessness-related words compared with non-words for **(A)** high and low symptom load groups separately, and (B) their contrast. MFG, middle frontal gyrus; IFG, inferior frontal gyrus; OFC, orbitofrontal cortex; PCG, paracingulate gyrus; SFG, superior frontal gyrus; SMG, supramarginal gyrus; PCC, posterior cingulate cortex; ACC, anterior cingulate cortex; MTG, middle temporal gyrus; COPE, contrast of parameter estimate. **(A)** The IFG, MFG and temporal pole demonstrated significant activity in the high symptom group, while in the low symptom group, the PCG, anterior insular, temporal pole, precuneus, PCC, ACC, SMG, thalamus SFG, MFG and IFG all demonstrated significant activity. For both high and low symptom groups, significant regions are displayed with a non-parametric threshold-free cluster enhancement *p* < 0.05. **(B)** The contrast of the two groups revealed significant regional activity in the low symptom group compared with the high symptom group in the anterior insula cortex with a non-parametric *p* < 0.05. **(C)** Mean COPE in arbitrary units (AU) for the high and low symptom load groups separately with standard deviation bars showing the variation in parameter estimates across participants (Finnegan et al., 2021).

COPD also alters cerebral function, largely driven by chronic hypoxia and respiratory dysfunction. Hypoxemic COPD patients exhibit greater anterior cerebral hypoperfusion and accelerated cognitive decline compared to non-hypoxemic patients (Antonelli Incalzi et al., 2003; Ortapamuk and Naldoken, 2006). Magnetic resonance spectroscopy studies indicate compensatory shifts in intracellular pH and disrupted cerebral phospholipid membranes in response to chronic hypoxia, alongside reduced metabolites in frontal and partial WM (Hamilton et al., 2003; Karakas et al., 2013). Transcranial magnetic stimulation methods indicated patients during acute exacerbation of COPD had less excitable motor cortex and higher corticospinal inhibition (Mohamed-Hussein et al., 2007; Alexandre et al., 2020). Rs-fMRI studies show widespread disturbance in intrinsic brain connectivity networks, particularly in the basal ganglia, where abnormal amplitude of low-frequency fluctuations (ALFF) correlates with partial pressure of oxygen (PaO_2_) (Dodd et al., 2012; Zhang J. et al., 2016; Lu et al., 2019). Network analyses reveal decreased global connectivity strength and nodal efficiency, predominantly in the visual and frontoparietal networks (Spilling et al., 2019; Wang et al., 2020). Additionally, frequency-dependent neural changes reveal ALFF abnormalities associated with arterial partial pressure of carbon dioxide (PaCO_2_) (Yu et al., 2021). Dynamic functional network connectivity studies show COPD patients remain in weakly connectivity longer than healthy controls, with aberrant connectivity primarily involving the default mode network, executive control network, and visual network. Correlation analysis showed dFNC indictors are correlate with FEV1, FEV1/FVC, and PaCO_2_ (Tang et al., 2022). Reduced motor cortical connectivity interactions with the brainstem and a shift in network dominance from the medulla to the motor cortex, potentially contributing to respiratory failure. These findings suggest therapeutic strategies to enhance respiratory muscle performance by modulating motor cortex (Yu et al., 2016). Metrics such as regional homogeneity (ReHo) and degree centrality (DC) confirm disrupted functional organization, especially in visual, motor, and default mode network (Xin et al., 2019; Li et al., 2020). ReHo values in the precuneus positively correlate with FEV1% (*r* = 0.465, *P* = 0.045), FEV1/FVC (*r* = 0.763, *P* < 0.001), while negatively correlate with PaCO_2_ (*r* = -0.611, *P* = 0.005) (Figure 5). The anterior insula plays a key role in modulating dyspnea perception in COPD patients. Greater activity was observed in patients with lower symptom burdens. This highlights the need for a multimodal approach to understanding dyspnea (Figure 6; Finnegan et al., 2021). COPD patients have impaired cerebrovascular reactivity (CVR) in anterior and posterior cerebral circulation. Impairment of CVR increase with the airflow limitation severity (Hlavati et al., 2019).

### 2.3 Cognitive effects of COPD on the brain

COPD is associated with the cognitive impairment, either globally or in specific cognitive domains, with its severity influenced by disease severity, arterial oxygen saturation and aging (Schou et al., 2012; Cleutjens et al., 2014; Yin et al., 2016; Samareh Fekri et al., 2017; Kakkera et al., 2018). Commonly affected cognitive domains include attention, executive function, working memory, and mental speed, reflecting prefrontal deficits in information integration than storage (Areza-Fegyveres et al., 2010; Lv et al., 2020). Hypoxia-induced cognitive impairments primarily affect memory and attention allocation, with memory deficits linked to limbic system involvement and attention issues attributed to diminished resource allocation (Stuss et al., 1997). Visuospatial construction impairments correlate with cortical thinning in the fronto-parietal network, driven in part by oxygen desaturation, contributing to deficits in visual memory and drawing tasks (Chen et al., 2016). GM reductions in regions related to dyspnea processing, fear regulation, and antinociception, are partially linked to disease duration and disease-specific fears, which may exacerbate the disease trajectory (Esser et al., 2016). Increased WML volume is associated with poorer episodic memory in stable COPD patients (Spilling et al., 2017). Functional changes in the DMN highlight key roles of the left posterior cingulate cortex and left hippocampus in cognitive decline (Hu et al., 2018). Dyspnea-related cognitive interference worsens motor control and daily functioning, long-term oxygen therapy may benefit neuropsychological and neurovascular function in mildly hypoxemic patients, its effects often lack statistical significance (Hjalmarsen et al., 1999; Hoiland et al., 2018; Kozora et al., 1999; Rozenberg et al., 2024). Integrating cerebral small vessel disease burden scores with cognitive scales could improve early detection of cognitive decline (Li et al., 2024a). COPD patients tend to outperform those with mild Alzheimer disease (AD) in neuropsychological tests, potentially due to relatively preserved brain network efficiency (Spilling et al., 2019). However, COPD exacerbates cognitive and mood disturbances in AD patients, highlighting the need for multidisciplinary management (Kozora et al., 1999; Schou et al., 2012; Tondo et al., 2018; de Kort et al., 2024). Mental health impairments, including anxiety, depression, and cognitive problems, influence the disease’s trajectory. Higher values in FEV1% predicted and FVC% predicted were associated with better global cognitive function in the cross-sectional analyses (Xiao et al., 2022). Comprehensive interventions that go beyond lung rehabilitation to incorporate mental health and cognitive support is crucial (Pelgrim et al., 2019).

## 3 Neuroimaging changes due to lung cancer

Lung cancer, including small cell lung cancer (SCLC) and non-small cell lung cancer (NSCLC), causes notable alterations in brain structure and function. Advanced neuroimaging has revealed these alterations. These changes contribute to cognitive and emotional impairments and highlight the neuropathological impact of the disease and its treatments.

### 3.1 Structural effects of lung cancer on the brain

SCLC patients underwent chemotherapy and prophylactic cranial irradiation (PCI) experience notable reductions in GM volume in the right subcortical regions, bilateral insular cortex, and superior temporal gyrus. Longitudinal assessments identified additional GM reductions in the right para-hippocampal gyrus and hippocampus, accompanied by widespread WM microstructural changes, particularly in the corpus callosum (Simó et al., 2016a). VBM and tract-based spatial statistics (TBSS) analyses corroborate these findings, showing decreased GM density in the bilateral basal ganglia, thalamus, and right insula, as well as WM microstructural abnormalities in the entire corpus callosum (Simó et al., 2016b). These findings underscore the neurotoxic impact of PCI on brain integrity. In addition to PCI, chemotherapy induces substantial brain changes in SCLC patients. Significant alterations in brain volume, cortical thickness, FA values, and functional connectivity measures were observed. DTI combined with graph theory revealed the topological reorganization of hub distributions in impaired brain structural network (Mentzelopoulos et al., 2021; Mentzelopoulos et al., 2022). Extensive WM microstructural damage has been identified in lung cancer patients with cancer pain. Disruption of specific nodes along pain-related fiber bundles were noted, these alterations may serve as sensitive imaging biomarker to characterize the severity and duration of cancer pain (Ran et al., 2024).

NSCLC patients experience brain network disruptions linked to cognitive and emotional deficits. Reductions in global efficiency are particularly evident in the left inferior frontal gyrus and right Rolandic area, with diminished local efficiency in the left middle frontal gyrus and left superior temporal gyrus. These alterations highlight the disruption of topological brain network features may underlie cognitive and emotional impairment in NSCLC patients (Liu et al., 2022c). Immune checkpoint inhibitor therapy in NSCLC patients is associated with detectable brain MRI abnormalities. These include stroke, typical WM lesions, and T2 hyperintensities indicative of central nervous system vasculitis or encephalitis. Interestingly, patients with such brain changes exhibited higher clinical benefit rates, longer progression-free survival, and a trend toward improved overall survival, suggesting a potential prognostic role for these imaging findings (Ni et al., 2022).

Radiomics provides non-invasive insights into brain metastases and molecular features. Textural features on MRI images may help to discriminate brain metastases of different primary tumors and guide treatment planning (Béresová et al., 2018; Li et al., 2024b). The SVM model integrating intra-tumoral and peritumoral radiomics features was confirmed as an imaging biomarker. It predicts brain metastases (BM) in newly diagnosed lung cancer patients. This highlights its potential to impact clinical diagnosis and treatment (Yichu et al., 2024). Deep learning methods combined with multiparametric MRI can also differentiate pathological subtypes of BM in lung cancer patients (Li et al., 2024c). Deep learning model integrating brain metastasis radiomics, clinical features, and a prognostic index provided reliable multi-time-point progression-free survival predictions for patients with advanced NSCLC and brain metastases (Wang et al., 2024).

### 3.2 Functional effects of lung cancer on the brain

**FIGURE 7:**
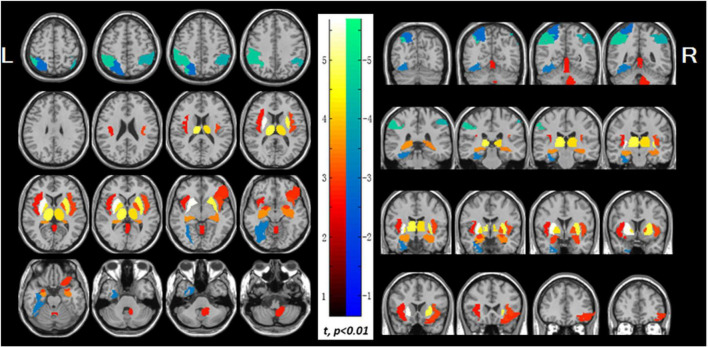
Abnormal glucose uptake in the non-small cell lung cancer patients. The increased regions display with red-to-white color, which include both left and right sides of the insula, putamen, pallidum, thalamus, hippocampus and amygdala, the right side of cerebellum, orbital part of right inferior frontal gyrus and vermis, while the decreased regions display with blue-to-green color, which include left superior parietal lobule, bilateral inferior parietal lobule, and left fusiform gyrus (*p* < 0.01). PET findings were overlaid on magnetic resonance image secondary. Color bar indicates *t*-values; L, left; R. right (Zhang W. et al., 2016).

Patients with lung malignancy showed a higher right cerebellar metabolism, potentially reflecting compensatory mechanisms to maintain respiratory and immune homeostasis (Golan et al., 2009). NSCLC patients underwent ^18^F-FDG PET prior to oncotherapy showed hypermetabolism in visceral-to-brain signal transduction pathways, while hypometabolism in dorsal attention network and visuospatial function areas (Figure 7). These changes may be associated with visceral sympathetic activation induced by lung cancer (Zhang W. et al., 2016). Post-PCI ^18^F-FDG PET studies in SCLC patients demonstrated significant decreases in glucose metabolism in the basal ganglia, central regions, cingulate cortex, striatum, frontal cortex, parietal cortex, occipital cortex, precuneus, lateral temporal cortex, and cerebellum (Chammah et al., 2021). These changes, further corroborated by textural analysis, may reflect radiation-induced damage, and provide novel approaches to study cognitive impairment (Sawyer et al., 2021). Lung function indices (FVC and FEV1) correlate with decreased standardized uptake value ratios in brain regions suggest that impaired lung function may exacerbate reduction in cerebral glucose metabolism and cognitive performance (Son et al., 2023).

**FIGURE 8:**
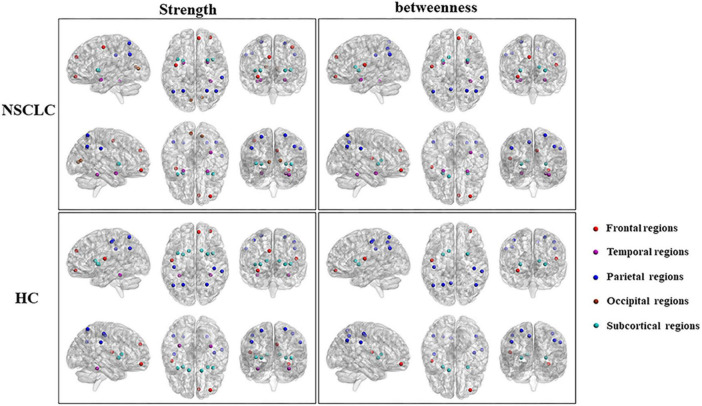
Differences in the distribution of hub regions between NSCLC and HC. Nodes with high nodal strength were classified as hub regions, which had intense interconnectivity with other regions in the brain and played an important in the information transfer and integration. Eleven of these regions [left and right superior parietal gyrus, putamen, and insula; left anterior cingulate gyrus; right middle frontal gyrus (orbital part), superior frontal gyrus (medial), fusiform gyrus, and supramarginal gyrus] were shared by both the NSCLC and HC groups. The NSCLC group lost hub properties in the left and right postcentral gyrus, caudate nucleus, left rolandic operculum, and fusiform gyrus, while the left and right amygdala, calcarine fissure, left precentral gyrus, and right anterior cingulate gyrus acted as new hub regions in the patient group. Nodes with high nodal betweenness were also classified as hub regions, which lied on the shortest path between two other regions and played an important role in controlling information flow. Nine of these regions (left and right anterior cingulate gyrus and putamen; left rolandic operculum, precuneus, and caudate nucleus; right middle frontal gyrus (orbital part) and supramarginal gyrus) were shared by two groups. The left and right amygdala, right superior frontal gyrus (medial), fusiform gyrus and superior parietal gyrus were defined as hub regions only in the NSCLC group. In the left and right postcentral gyrus, left superior parietal gyrus was defined as hub regions only in HC. Strength, nodal strength; betweenness, nodal betweenness; NSCLC, non-small cell lung cancer; HC, healthy control (Liu et al., 2022b).

Rs-fMRI analyses revealed lung cancer patients exhibited reduced rs-fMRI values in frontal and parietal regions before and after chemotherapy (You et al., 2020). Reduced dynamic activity in DMN after chemotherapy suggests its susceptibility to treatment-related damage. Decreased FC in posterior cingulate cortex (PCC) and anterior cingulate cortex (ACC) correlates with reduced MoCA, implicating DMN disruptions in chemotherapy-induced cognitive impairment (Zhang et al., 2020). Graph-theoretical analyses identify topological abnormalities in the pallidum-thalamus-cortex circuitry (see Figure 8). NSCLC patients show altered hub strength in key brain regions. Strength reduced in the inferior frontal gyrus but increased in the pallidum and thalamus. Hub mediation is also reduced in the superior occipital gyrus. These topological abnormalities in may reflect the pathological mechanisms of NSCLC and serve as potential biomarkers (Liu et al., 2022b). Dynamic functional connectivity analyses further identified deficits across sensorimotor, attention and auditory networks, emphasizing the extensive impact of chemotherapy on the brain resting-state network (Hu et al., 2022).

Patients with bone metastases from lung cancer (BMP) exhibit disrupted intrinsic brain connectivity. Reduced ALFF and ReHo were observed in the prefrontal cortex, alongside increased ReHo in the bilateral thalamus and left fusiform gyrus, with decreased FC within the prefrontal cortex (Liu et al., 2022a). In patients with cancer pain (CP+), hypo-connectivity is observed across somatomotor, ventral attention, fronto-parietal control, and DMN compared to controls. However, CP+ patients uniquely demonstrate hyper-connectivity in somatomotor and visual networks, potentially reflecting neural adaptations to pain. Increased connectivity strength within and between network modules correlates strongly with pain intensity and duration, particularly involving regions such as the dorsolateral prefrontal cortex, ACC, secondary somatosensory cortex, and amygdala (Wei et al., 2024; Zhou X. et al., 2022). Altered functional activity and connectivity in the prefrontal cortex, fusiform gyrus, and thalamus may be relevant to the neuropathology of BMP and is expected to be a potential biomarker for predicting BMP patients (Liu et al., 2022a).

### 3.3 Cognitive effects of lung cancer on the brain

SCLC patients underwent chemotherapy and PCI exhibited cognitive deterioration. This included verbal fluency decline, with nearly half meeting criteria for cognitive impairment. Cognitive decline correlated with increased mean FA values in the corpus callosum (Simó et al., 2016a; Simó et al., 2016b).

The structural and functional brain changes in lung cancer patients underscore the profound impact of cancer and its treatments on neural integrity. These findings highlight the importance of incorporating advanced neuroimaging techniques into clinical practice to monitor brain health, mitigate cognitive and emotional deficits. Further research is warranted to elucidate the mechanisms underlying these changes and to explore potential protective strategies.

## 4 COVID-19 and neuroimage changes

### 4.1 Structural effects of COVID-19 on the brain

**FIGURE 9:**
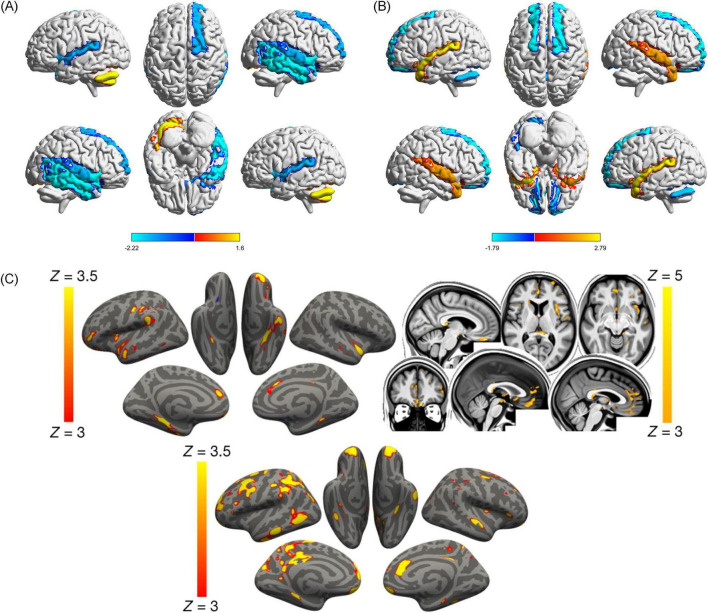
Cerebral structural and functional changes on COVID-19 patients. **(A)** COVID-19 patients displayed decreased functional activity in the right superior temporal gyrus (STG) [extending to the right middle temporal gyrus [MTG], insula, inferior temporal gyrus [ITG], and temporal pole (TP)], left insula, right orbitofrontal cortex (OFC) (extending to the right olfactory cortex), and increased functional activity in left cerebellum (Guo et al., 2024). **(B)** Compared with HCs, COVID-19 patients displayed decreased GMV in the bilateral anterior cingulate cortex/ medial prefrontal cortex (ACC/ mPFC) (extending to the bilateral OFC), and left cerebellum, and increased GMV in the bilateral amygdala (extending to the bilateral hippocampus, STG, TP, and MTG, and left striatum) (Guo et al., 2024). In **(A,B)** Red areas represent increased functional activity or GMV value, and blue areas represent decreased functional activity or GMV value. SDM-Z values are shown in color on the color bar. COVID-19, coronavirus disease 2019; HCs, healthy controls; SDM, seed-based mapping; GMV, gray matter volume (Guo et al., 2024). **(C)** Vertex-wise and voxel-wise longitudinal effects on COVID-19 patients. Top, the thresholded map (| Z| > 3) shows that the strongest, localized reductions in gray matter thickness in the infected participants compared with the controls are bilaterally in the parahippocampal gyrus, anterior cingulate cortex, and temporal pole, as well as in the left orbitofrontal cortex, insula and supramarginal gyrus. The strongest longitudinal differences in mean diffusivity (| Z| > 3, left is shown on the right) could be seen in the orbitofrontal cortex and anterior cingulate cortex, as well as in the left insula and amygdala (top). Bottom, the thresholded cortical thickness map (| Z| > 3) demonstrated longitudinal differences between the hospitalized and non-hospitalized COVID-19 cases in the orbitofrontal frontal cortex and parahippocampal gyrus bilaterally, right anterior cingulate cortex, as well as marked widespread differences in fronto-parietal and temporal areas, especially in the left hemisphere (Douaud et al., 2022).

COVID-19 has been linked to macro- and microstructural brain changes, as demonstrated through imaging studies. These changes affect both the central and peripheral nervous systems, with radiological findings revealing a wide spectrum of vascular and inflammatory effects (Lu et al., 2023; Klironomos et al., 2020; Kiyak et al., 2024). Brain MRI has revealed unusual distribution of microbleeds predominantly in the corpus callosum, along with infractions and intracranial hemorrhages in the thalamus and corpus callosum (Fitsiori et al., 2020; Klironomos et al., 2020; Kelsch et al., 2021). DTI study revealed increased mean diffusivity (MD) and radial diffusivity (RD) alongside decreased FA in WM tracts, including the corpus callosum, coronal radiations, and superior longitudinal fasciculus (Mohammadi and Ghaderi, 2024). Network diffusion modeling suggests that pathologic changes spread across the corticospinal tracts, cerebellum, and putamen, indicating structured propagation of neurological injury (Parsons et al., 2021). Structural and functional changes are observed in the temporal lobe, orbitofrontal cortex, and cerebellum. Additional disruptions were noted in the insula and limbic system, with specific alterations in the right superior temporal gyrus showing reduced functional activity but increased GM volume (Figures 9A, B; Guo et al., 2024). However, genetic susceptibility to COVID-19 showed no causal association with structural brain changes (Ding and Xu, 2023).

Longitudinal MRI studies reported progressive reductions in GM thickness, particularly in the orbitofrontal cortex and para-hippocampal gyrus, along with global brain atrophy (Douaud et al., 2022; Figure 9C). Critically ill patients recovering from COVID-19 displayed complications during follow-up, including GM atrophy and altered WM diffusivity in cortical, limbic, and cerebellar regions, which correlated with cognitive dysfunction (Lersy et al., 2022; Díez-Cirarda et al., 2023). COVID-19 survivors exhibited cortical morphological network changes across multiple regions, this alteration reflects the extensive brain structural remodeling triggered by COVID-19 (Long et al., 2023). While longitudinal imaging data from multiple sclerosis patients suggested no acceleration of global GM atrophy after COVID-19 infection, but regional trends, such as reduced para-hippocampal gyrus volume (Rebsamen et al., 2023). Structural MRI studies in post-COVID conditions (PCCs) consistently reported gray matter volume reduction and cortical thickness in both cerebral cortical and subcortical regions. Long-term COVID-19 patients showed lower MD values in internal capsule, anterior and posterior coronal radiations, and corpus callosum, but the effect sizes were minimal. Despite reported neuropsychiatric symptoms, no significant differences in objective cognitive performance were observed (Nelson et al., 2024). These findings contribute to the understanding of the acute and chronic effects of the virus on the nervous system and potentially inform acute and long-term treatment and neurorehabilitation decisions.

### 4.2 Functional effects of COVID-19 on the brain

**FIGURE 10:**
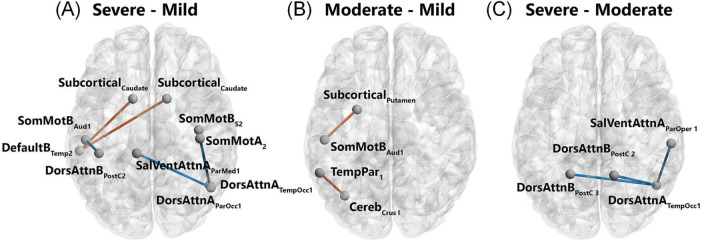
Patterns of significantly different functional connectivity in the COVID-19 patients. Differences in functional connectivity between brain structures shown in a network representation when comparing severe versus mild **(A)**, moderate versus mild **(B)**, and severe versus moderate **(C)**. Blue lines indicate a decrease in the connectivity measurement (mean decrease = -0.3), red lines indicate an increase in the connectivity measurement (mean increase = 0.3). Statistical significance was false discovery rate (FDR)-corrected for multiple comparisons (*p* < 0.05 FDR). Networks: Cereb, cerebellum; DefaultB, default mode B; DorsAttnA and DorsAttnB, dorsal attention A and B; SalVentAttA, salience ventral attention A; SomMotA and SomMotB, somatosensory motor A and B; TemPar, temporoparietal; Regions: Aud, auditory cortex; ParMed, parietal medial; ParOcc, parietal occipital cortex; ParOper, parietal operculum; PostC, postcentral region; Temp, temporal region; TempOcc, temporo-occipital region (Voruz et al., 2023).

Functional neuroimaging studies have revealed widespread disruptions in brain activity and connectivity among COVID-19 patients. Static amplitude of low-frequency fluctuation (sALFF) was reduced in the right lingual gyrus and left medial occipital lobe, whereas dynamic ALFF (dALFF) increased in the right rectus gyrus. Static functional connectivity (sFC) was diminished between the lingual gyrus and the right superior occipital lobe, while dynamic functional connectivity (dFC) was reduced in the anterior central gyrus. Integrating dynamic and static ALFF and FC metrics into a support vector machine (SVM) model enabled high-accuracy identification of COVID-19 patients (Han et al., 2024). Functional connectivity studies highlight extensive network disruptions, particularly in critically ill patients. These patients exhibit both low and high FC patterns, whereas those with moderate illness predominantly show high FC patterns involving networks such as DMN, sensorimotor, dorsal attention, subcortical, and cerebellar networks (Figure 10; Voruz et al., 2023). COVID-19 patients with rehabilitation syndrome present hypoconnectivity between the orbitofrontal and cerebellar regions bilaterally, altered functional brain connectivity in the para-hippocampal gyrus (Díez-Cirarda et al., 2023).

Metabolic imaging findings further corroborate these functional disruptions. FDG-PET imaging revealed hypometabolic patterns, suggesting network-level dysfunction in some long-term COVID-19 patients (Verger et al., 2022a; Verger et al., 2022b). Arterial spin labeling MRI revealed significant hypoperfusion in patients with subjective cognitive deficits, mainly affecting the prefrontal, parietal, and temporal lobes in the right hemisphere (Ajčević et al., 2023). Perfusion-weighted imaging confirmed reduced cerebral blood flow (CBF) in anterior temporal regions, thalamus, and basal ganglia among PCCs patients (Mohammadi and Ghaderi, 2024). Whole-brain and WM CBF reductions in mildly affected individuals were predictive of COVID-19 status (85.2% accuracy), suggesting that CBF patterns may be potential imaging markers (Sen et al., 2023).

### 4.3 Cognitive effects of COVID-19 on the brain

Neuroimaging findings align with observed cognitive impairments in COVID-19. Cognitive and structural brain changes in hospitalized patients suggest a relationship between the severity of COVID-19 and the extent of neurocognitive impact (Díez-Cirarda et al., 2023). Critically ill patients exhibit functional deficits in verbal memory, while moderate patients show reduced mental flexibility, correlation analyses confirm specific associations between memory, executive function performance and altered FC (Voruz et al., 2023). The cognitive changes may result from a combination of neurodegenerative processes, neuroinflammation and sensory deprivation through olfactory pathway. These mechanisms likely contribute to the *in vivo* markers of gray matter volume (GMV) reduction in limbic system (Douaud et al., 2022).

## 5 Neuroimaging changes in other lung diseases

Neuroimaging studies have also revealed brain alterations in various lung diseases, closely associated with cognitive, emotional, and functional impairments, underscoring the importance of integrating neurological assessments into lung disease management.

WM abnormalities are observed in infants receiving mechanical ventilation and chronic lung disease patients (Anjari et al., 2009), including bronchopulmonary dysplasia (Lee J. M. et al., 2019), whereas improved respiratory morbidity is associated with WH microstructure recovery (Ball et al., 2010). Survivors of acute respiratory distress syndrome (ARDS) demonstrated cognitive impairments associated with brain atrophy, ventricular enlargement, highlighting the long-term neurological sequelae of ARDS (Hopkins et al., 2006). Asthmatics exhibited enhanced functional connectivity in the DMN and salience network, correlating with higher depression, anxiety, and poor asthma control. Rs-fMRI revealed abnormalities in the ventral anterior insula linked to depression in asthma. Percent amplitude of fluctuation analysis identified altered activity in brain regions governing respiration, memory, language, and attention. In children, impairments in the superior frontal gyrus and parietal lobe were associated with attention deficits, underscoring the impact of asthma on cognitive function across age groups (Zhang et al., 2017; Wang et al., 2023; Zhu et al., 2023). Lung arterial hypertension (PAH) patients demonstrate reduced GMV in hippocampus, insula, cerebellum, and frontal, temporal, parietal, and occipital lobes. Additionally, elevated T2 relaxation values were observed in the cerebellum, hippocampus, and frontal lobe. These neuroanatomical changes may contribute to tissue damage and cognitive deficits in PAH patients (Roy et al., 2021). Genetic predispositions in idiopathic lung fibrosis patients were associated with alterations in cortical morphology and WM microstructure (Mohammadi-Nejad et al., 2022). Reduced gray matter volume in the frontal, parietal, and temporal lobes, as well as the anterior cingulate gyrus, insula, and cerebellar cortex, were observed in lung transplant patients. These changes are thought to contribute to perioperative cognitive deficits, emphasizing the need for strategies to protect brain tissue during and after transplantation (Vandiver et al., 2022). Rs-fMRI studies in chronic cough patients revealed reduced FC between the nuclear solitary tract and anterior cingulate cortex, which may contribute to cough hypersensitivity and anxiety in chronic cough patients (Wu et al., 2023).

## 6 Mechanisms of neuroimaging findings in lung diseases

Hypoxia and brain damage: Hypoxia reduces brain metabolism and increases oxidative stress, leading to structural and functional damage. COPD and COVID-19 show similar mechanisms of hypoxic brain damage, particularly in the prefrontal cortex, hippocampus, and subcortical structure. Hypoxia leads to volume reduction and metabolic decline in these areas, making them particularly vulnerable to cognitive decline and memory loss. Functional imaging shows that hypoxia causes decreased metabolic activity in brain regions associated with memory, mood, and executive function, particularly in COPD patients. In COVID-19 patients, hypoxia due to respiratory failure exacerbates damage in the frontal and temporal lobes, with cerebral metabolism gradually recovering but remaining below normal levels during recovery.

Neuroinflammation and cerebrovascular damage: Neuroinflammation can lead to microstructural changes in the brain. In lung cancer patients undergoing PCI and chemotherapy, cerebrovascular damage is often observed as widespread WM hyperintensities on imaging. MRI and PET studies have also revealed the extensive inflammatory response associated with COVID-19 may disrupt the blood-brain barrier, leading to substantial neural tissue injury. Additionally, growing studies highlight a strong link between COVID-19, chronic lung diseases, and cerebrovascular structural damage, manifesting as microbleeds, thrombosis, and microinfarcts. These pathologies are thought to be driven by systemic inflammation and dysregulated coagulation process associated with lung diseases.

## 7 Clinical significance of neuroimage research

Early identification of brain damage: Neuroimaging detects early brain changes in COPD and COVID-19 patients. These changes, often seen before cognitive decline manifests, facilitating timely intervention to minimize neurological damage (Xiao et al., 2022; Zhou L. et al., 2022; Zhang et al., 2013; Yin et al., 2019; Fitsiori et al., 2020; Klironomos et al., 2020; Kelsch et al., 2021; Mohammadi and Ghaderi, 2024; Verger et al., 2022a; Verger et al., 2022b; Ajčević et al., 2023). For the brain regional changes identified at early stage, targeted neuromodulation can be applied through techniques such as transcranial electrical stimulation (tES) or transcranial magnetic stimulation (TMS) as part of personalized intervention strategies.

Prediction of cognitive impairment: Structural brain changes, particularly in the prefrontal cortex and hippocampus, are strongly linked to cognitive decline in COPD patients. Brain imaging helps predict cognitive dysfunction, guide cognitive interventions, and monitor rehabilitation programs. Moreover, neuroimaging helps to assess the neurotoxic effects of PCI and chemotherapy, allowing for more tailored therapeutic approaches (Schou et al., 2012; Cleutjens et al., 2014; Yin et al., 2016; Samareh Fekri et al., 2017; Kakkera et al., 2018; Stuss et al., 1997; Simó et al., 2016a; Simó et al., 2016b).

Assessing the effects of hypoxia on brain function: Chronic hypoxia in COPD contributes to brain atrophy and cognitive decline. Neuroimaging studies reveal that hypoxia leads to metabolic and perfusion changes in the brain, further exacerbating these impairments. Thus, monitoring and intervening in hypoxic conditions is essential for preserving brain function and delaying cognitive deterioration (Antonelli Incalzi et al., 2003; Ortapamuk and Naldoken, 2006; Hamilton et al., 2003).

Optimizing treatment strategies: Neuroimaging evaluates the treatment impacts on brain structure and function. Long-term oxygen therapy has shown potential in mitigating neuropsychological decline, and brain imaging can track its effectiveness. Neuroimaging identifies early signs of brain injury in COVID-19, which can inform the development of targeted interventions, including anti-inflammatory therapies to reduce neurodegeneration (Hjalmarsen et al., 1999; Hoiland et al., 2018; Mohammadi and Ghaderi, 2024; Sen et al., 2023; Douaud et al., 2022). Combined with imaging histology and deep learning techniques, neuroimaging can predict molecular features and treatment outcomes, provide scientific basis to understand the mechanisms of neurotoxicity and adaptations in brain function associated with lung cancer treatment. This can be used to develop pharmacological or non-pharmacological neuroprotective strategies (Li et al., 2024c; Wang et al., 2024; Li et al., 2024b; Ho et al., 2019).

Support for mental health interventions: Anxiety and depression are prevalent in COPD patients and can exacerbate cognitive deficits. Neuroimaging identifies brain regions affected by these conditions, aiding interventions to improve both mental health and cognitive function (Pelgrim et al., 2019; Heck et al., 2022; Golan et al., 2009; Wei et al., 2024; Zhou X. et al., 2022).

## 8 Limitations of current research and future directions

### 8.1 Shortcoming of current studies

Small sample size: many studies involve limited cohorts, compromising the reliability and stability of statistical conclusions.

Non-standardized research criteria: The research criteria for different lung diseases are not standardized. Correlation without causation: Most studies can only perform correlation analysis, which makes it difficult to determine the causal relationship between lung disease and brain changes.

Lack of longitudinal studies: Lack of follow-up studies that follow patients over time to assess chronic and progressive changes in the effects of lung disease on the brain.

Absence of reliable biomarkers: And reliable biomarkers are still needed to predict and monitor the effects of lung disease on the brain.

### 8.2 Suggests possible directions for future research

Large-scale, multicenter studies: recruiting more patients with multicenter studies improves the generalizability and reliability of results.

Artificial intelligence and machine learning: Leveraging machine learning to identify imaging biomarkers and explore the relationship between lung disease progression and cognitive function. For example, convolutional neural networks (CNNs) or Transformer models can be used for disease classification. Model interpretability studies can help identify biomarkers that have a positive impact on the model in disease classification. Deep unsupervised clustering models can be applied to analyze different subtypes of the same disease, providing insights into diverse disease manifestation patterns. Graph neural networks (GNNs) can be employed to analyze the causal relationships between lung diseases and brain structural changes. Causality verification: Employ longitudinal tracking and causal inference models to elucidate the bidirectional relationship between lung disease and brain changes, including whether neural abnormalities may predispose individuals to lung conditions.

Neuromodulation and autonomic nervous system: Investigating the role of brain centers in respiratory regulation and prefrontal cortex influence on the autonomic nervous system could clarify mechanisms underlying the lung-brain axis. Lung rehabilitation as a multidisciplinary strategy can improve physical function and quality of life by improving respiratory and limb motor control through neuroplasticity. Brain activity associated with breathlessness predicts improvements in symptoms. Future studies could increase imaging studies of neuromodulation and analyze whether functional changes in region of neuromodulation influence susceptibility to and progression of lung disease.

Systemic impacts of respiratory disease: understanding how autonomic nervous system dysregulation triggered by lung disease cascades into systemic effects, may reveal indirect pathways contributing to chronic disease progression.

## 9 Conclusion

Neuroimaging provides critical insights into the complex relationship between lung disease and brain. It enables early detection of brain structural and functional alterations, which can guide the management of cognitive impairments in COPD, lung cancer, and COVID-19 patients. Additionally, neuroimaging can identify specific brain regions affected by disease or treatment, helping to optimize therapeutic interventions. COPD primarily affects the frontal lobes, with gray matter atrophy and reduced perfusion. COVID-19 exhibits widespread microhemorrhages and neuroinflammation, particularly in regions functionally connected to the olfactory cortex. Lung cancer-related changes are often linked to the neurotoxicity of radiotherapy and chemotherapy. The affected regions are concentrated in subcortical structures, while cancer pain is associated with hyperconnectivity in motor and visual networks. The findings underscore the importance of integrating advanced neuroimaging techniques into clinical practice, offering personalized approaches for monitoring cerebral health and improving the management of lung diseases. Further research is needed to explore the mechanisms behind these brain changes and develop neuroprotective strategies to mitigate treatment-related brain damage.
