# Correction: Neuroimaging insights into lung disease-related brain changes: from structure to function

**DOI:** 10.3389/fnagi.2025.1738176

**Published:** 2025-11-19

**Authors:** Zhixi Zhang, Miao He, Yubo Liu, Zhongtian Guan, Chunlin Li

**Affiliations:** 1 School of Biomedical Engineering, Capital Medical University, Beijing, China; 2 Aerospace Information Research Institute, Chinese Academy of Sciences, Beijing, China

**Keywords:** lung-brain axis, neuroimaging, lung disease, MRI-based brain changes, neurological impact of lung disease

Affiliation School of Biomedical Engineering, Capital Medical University, Beijing, China was omitted from the paper. This affiliation has now been added for author Zhixi Zhang.

The order of authors in the author list of the published paper was erroneously given as:

Miao He^†^, Yubo Liu, Zhongtian Guan, Chunlin Li^*^, Zhixi Zhang^*^

^†^These authors have contributed equally to this work

The correct author list reads:

Zhixi Zhang^†^, Miao He^†*^, Yubo Liu, Zhongtian Guan, Chunlin Li^*^

^†^These authors have contributed equally to this work

Incorrect author assigned as corresponding author

Author Zhixi Zhang was erroneously assigned as corresponding author. The correct corresponding author is Miao He (email: hemiao@mail.ccmu.edu.cn).

Author Zhixi Zhang was erroneously omitted as equal contributing author.

The original version of this article has been updated.

