# Correction: Neuroimaging insights into lung disease-related brain changes: from structure to function

**DOI:** 10.3389/fnagi.2026.1923503

**Published:** 2026-07-13

**Authors:** Zhixi Zhang, Miao He, Yubo Liu, Zhongtian Guan, Chunlin Li

**Affiliations:** 1 School of Biomedical Engineering, Capital Medical University, Beijing, China; 2 Aerospace Information Research Institute, Chinese Academy of Sciences, Beijing, China

**Keywords:** lung-brain axis, neuroimaging, lung disease, MRI-based brain changes, neurological impact of lung disease

In the published article, the Acknowledgments section was erroneously omitted. The **Acknowledgments** section appears below:

We express our gratitude to all those who provided assistance. We also thank Liwei Sun from Capital Medical University for his assistance with the experimental paradigm, including advice on paradigm logic, stimulus materials, and audiovisual stimulus presentation.

In the published article, in the Funding section, the funder “Capital Medical University Clinical-Basic Research Collaborative Platform Development Project, [JLPYPT2025007]” was erroneously omitted. The corrected **Funding** statement appears below.

The author(s) declared that financial support was received for this work and/or its publication. This study was supported by Capital Medical University Clinical-Basic Research Collaborative Platform Development Project, [JLPYPT2025007].

The original version of this article has been updated.

